# Supplementary material for: The UTRs of Leishmania donovani vary in length and are enriched in potential regulatory structures
Source: bioRxiv. 2025 Sep 22:2025.09.22.677690. Preprint. [Version 1] doi: 10.1101/2025.09.22.677690 (PMC12485950; doi:10.1101/2025.09.22.677690)
Supplement: Supplement 1 [file NIHPP2025.09.22.677690v1-supplement-1.pdf]

bioRxiv preprint doi: https://doi.org/10.1101/2025.09.22.677690; this version posted September 22, 2025. The copyright holder for this preprint (which was not certified by peer review) is the author/funder, who has granted bioRxiv a license to display the preprint in perpetuity. It is made available under aCC-BY 4.0 International license.

Supp Figure 1: Individual Ld1S chromosome-specific Nucmer plot from chromosome that have been assembled by Flye (y-axis) in a single contig compared to BPK282A1 (x-axis). Red and blue blocks represent the different PTU on each chromosomes: blue blocks represent PTUs on the negative strand and red blocks PTUs on the positive strand.

Supp Figure 2: Individual Ld1S chromosome-specific Nucmer plot from chromosome that have been assembled by Flye (y-axis) in multiple contigs compared to BPK282A1 (x-axis). Red and blue blocks represent the different PTU on each chromosomes: blue blocks represent PTUs on the negative strand and red blocks PTUs on the positive strand.

Supp Figure 3: Representation of the chromosomal rearrangement between of Ld1S chromosome 11 (A), 13 (B), 18 (C), 28 (D) and 29 (E) (at the bottom) and BPK282A1 (at the top). Each arrow represents a gene; the ones in blue and red are not inverted or translocated, a vertical bar represents a transcription termination stop, and oppositive arrows represent transcription switches between two PTUs. Chromosomes 18 and 29 have partial or full polycistronic reads supporting the rearrangement.

Supp Figure 4: IGV screenshot showing misannotations due (A) to non-removal of a polycistron due to the presence of a non-coding RNA and (B) to interrupted read sequencing.

Supp Figure 5: (A) Comparison of promastigotes raw count per genes from all reads and SL/polyA filtered read.

bioRxiv preprint doi: https://doi.org/10.1101/2025.09.22.677690; this version posted September 22, 2025. The copyright holder for this preprint (which was not certified by peer review) is the author/funder, who has granted bioRxiv a license to display the preprint in perpetuity. It is made available under aCC-BY 4.0 International license.

Two points representing Ld1S.272530.1 (5.8S rRNA) and Ld1S.272550.1 (18S rRNA) with respectively 150,143, 53,245 and 19,021 raw reads and 40, 6 and 8 after filtering for SL and polyA tail were left out because they are off range.

Supp Figure 6: MEME motif search on the 50 first nucleotides of each ONT read.
Motif search on the 50 first nucleotides and comparison to *L. donovani* spliced-leader. Motif I and II were the most abundant in all ONT reads while motif III was enriched after using Fuzznuc to selected for the longest Spliced-Leader for the annotation.

Supp Figure 7: Average of the UTR length over the transcript length of the 5' UTR and 3'UTR is similar across chromosomes.
The blue boxes represent the distribution of the ratio of the 5' UTR length and the orange boxes the one of the 3' UTR

Supp Figure 8: Partitioning of gene expression variance across genomic features using fixed-effect linear modeling.
A linear model was used to quantify the proportion of variance in gene expression (log_2-transformed CPM) explained by gene length, position within the polycistronic transcription unit (PTU), and PTU identity (PTU ID), all modeled as fixed effects. Gene length and PTU ID account for 8.0% and 4.3% of the variance, respectively, while position in PTU contributes negligibly. The majority of variance (78.3%) remains unexplained by the model, likely reflecting additional biological and technical factors.

Supp Table 1: Quast genome quality table

Supp Table 2: NUCmer summary table of SNP differences between Ld1S and LdBPK282A1
P1 and P2, 1-based coordinates of the variant in the reference and query sequences, respectively; SUB, the reference and query characters at that site; BUFF and DIST, distances to the previous variant in the reference and query; LEN R and LEN Q, lengths of the aligned reference and query

bioRxiv preprint doi: https://doi.org/10.1101/2025.09.22.677690; this version posted September 22, 2025. The copyright holder for this preprint (which was not certified by peer review) is the author/funder, who has granted bioRxiv a license to display the preprint in perpetuity. It is made available under aCC-BY 4.0 International license.

sequences for that block; FRM, alignment orientation (1 = forward, −1 = reverse, printed as reference, query: and TAGS, the sequence IDs.

Supp Table 3: NUCmer summary table of DNA rearrangement between Ld1S and LBPK282A1 Columns are: SEQ, the Ld1S sequence ID; TYPE, event class; S1**/**E1, 1-based start and end coordinates on SEQ; LEN 1, event size on SEQ (negative values indicate reverse orientation). Event codes: BRK (alignment break), GAP (insertion/deletion between blocks), DUP (local/tandem duplication), INV (inversion), JMP (relocation/translocation "jump" between non-syntenic blocks), and SEQ (sequence-to-sequence junction reported by the aligner). Coordinates are reported in the coordinate system of SEQ; signs reflect orientation.

Supp Table 4: Summary table of transferred annotation for LdBPK282A1 including newly annotated lncRNA and monocistronic transcripts

Supp Table 5: Table of isoforms found in promastigotes and amastigotes
